# Supplementary material for: Absence of Dx2 at Glu-D1 Locus Weakens Gluten Quality Potentially Regulated by Expression of Nitrogen Metabolism Enzymes and Glutenin-Related Genes in Wheat
Source: Int J Mol Sci. 2020 Feb 18;21(4):1383. doi: 10.3390/ijms21041383 (PMC7073084; doi:10.3390/ijms21041383)
Supplement: Supplementary file 1 [file ijms-21-01383-s001.pdf]

Absence of Dx2 at *Glu-D1* Locus Weakens Gluten Quality Potentially Regulated by Expression of Nitrogen Metabolism Enzymes and Glutenin-Related Genes in Wheat

Lijun Song, Liqun Li, Liye Zhao, Zhenzhen Liu, Tingting Xie, and Xuejun Li*

College of Agronomy and State Key Laboratory of Crop Stress Biology in Arid Areas, Northwest A&F University, Yangling, Shaanxi 712100, China

Appendix A. Supplementary data

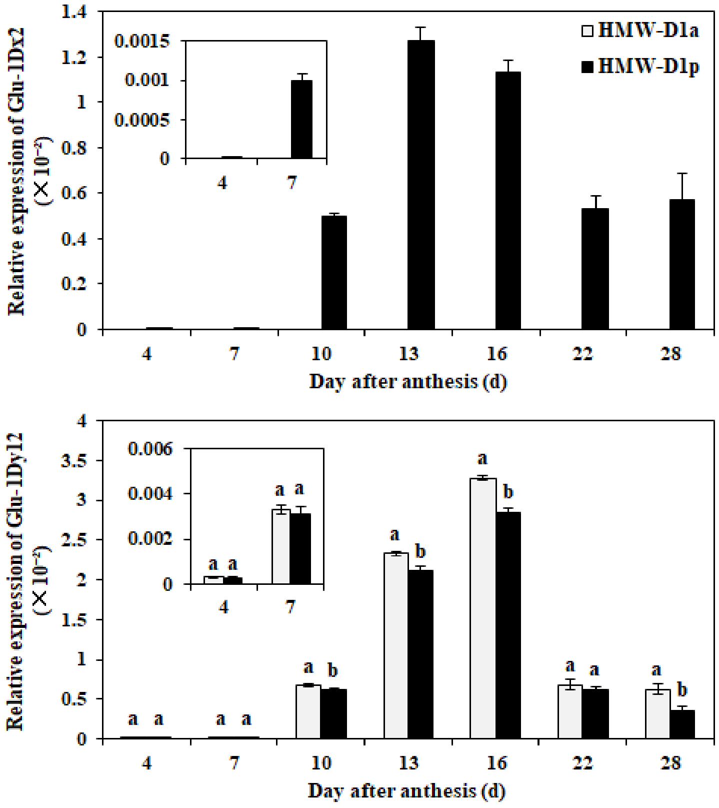

Fig. S1. Expression levels of *Glu-1Dx2* and *Glu-1Dy12* in HMW-D1a and HMW-D1p grains during the development in 2018–2019 growing season. Gray column represents HMW-D1a and black column represents HMW-D1p. Results followed by a different letter in the same column within the same group are significantly different ($P < 0.05$).

Table S1. List of primers used for quantitative real-time PCR analysis of all genes

Gene	Forward 5′→3′	Reverse 5′→3′
GS1	CGCCGACATCAACACCTTCAG	GTCCTCGAAGTAGCCCTTGCC
GS2	GGGCTACACGAGACAGCTAG	ATCCTTTGCCCTTTGCCTC
AlaAT	AATCCCTGCAATCATATCCAG	TCCTCCGCTTTCAATCGTAG
WCP2	CACCACATTGCCCTCCTCCC	CAGCGGCACTATGGTACGTC
TA.61026	ATCGCCAACAACTCAACGTAC	CCTCGCAGTTGGGTTTGAC
Dx2	GCAGCAACTCCAACAACGTA	GCCTGGATAGTATGAAACCTG
Dy12	CCCAAGCGTAACTTCTCCTCGG	TGTTGCCCTTGTCCTGGTTC
GAMyb	GGCACCTTCTCTACTTCTAGGAC	AGGAGCCACGGTATACTTGAG
SPA	ATTGACAATAGGGTACTAAAGGC	CTTGGGGGAAATTGTTGGTG
PBF	CATTCCAGTTCCGATGCAG	GTCATCTCCTTCCTCGCTAG
PDIL2-1	GCCCATCATTAAGGAAACAG	AAGTAATTGGCAACAGGTTC
PPIase	AAGAGCCATTTGAATTTAAGAC	GAATAGTGACAAGAGCAACC
SUMO1	TCCGCATCAAGAGATCCACAC	TCACCACGGAGCCTACGAC
18S	AACACTTCACCGGACCATTCA	CGTCCCTGCCCTTTGTACAC

AlaAT, alanine aminotransferase; Dx2 and Dy12, high molecular weight glutenin subunit 2 and 12; GAMyb, gibberellin-response MYB; GS1 and GS2, glutamine synthetase 1 and 2; PBF, prolamin-box binding factor; PDIL2-1, protein disulfide isomerase-like 2-1; PPIase, peptidyl-prolyl cis-trans isomerase; SPA, storage protein activator; SUMO1, small ubiquitin-related modifier 1; TA.61026 encoding thiol protease; WCP2, encoding cysteine protease.
